# Improved access to early diagnosis and complete treatment of malaria in Odisha, India

**DOI:** 10.1371/journal.pone.0208943

**Published:** 2019-01-02

**Authors:** Sreya Pradhan, Madan Mohan Pradhan, Ambarish Dutta, Naman K. Shah, Pyare Lal Joshi, Khageshwar Pradhan, S. K. Sharma, Penny Grewal Daumerie, Jaya Banerji, Stephan Duparc, Kamini Mendis, Shiva Murugasampillay, Neena Valecha, Anupkumar R. Anvikar

**Affiliations:** 1 National Vector Borne Disease Control Programme, Government of Odisha, Bhubaneswar, India; 2 Indian Institute of Public Health, Bhubaneswar, India; 3 Kalinga Institute of Industrial Technology, Deemed to be University, Bhubaneswar, India; 4 University of North Carolina, Chapel Hill, North Carolina, United States of America; 5 Independent Malariologist, New Delhi, India; 6 National Institute of Malaria Research Field Unit, Rourkela, India; 7 National Institute of Malaria Research, New Delhi, India; 8 Medicines for Malaria Venture, Geneva, Switzerland; 9 Independent Malariologist, Colombo, Sri Lanka; 10 Global Public Health, Geneva, Switzerland; Ministry of Health and Sports, MYANMAR

## Abstract

**Background:**

In 2013, the Comprehensive Case Management Programme (CCMP) was initiated to assess the impact of universal access to diagnosis and treatment and improved surveillance on malaria transmission in different settings in Odisha state, India.

**Methods:**

Pairs of intervention and control sub-districts (blocks), matched on malaria incidence were selected in four districts with different transmission intensities. CCMP activities included training and supervision, ensuring no stock-outs of malaria tests and drugs, analysing verified surveillance data, stratifying areas based on risk factors, and appointing alternative providers to underserved areas. Composite risk scores were calculated for each sub-centre using principal component analysis. Post−pre changes (2013–2015 versus 2011–2012) for annual blood examination rates (ABER) and annual parasite incidence (API) across intervention and control groups were assessed using difference-in-difference (DID) estimates, adjusted for malaria transmission risk.

**Results:**

In the intervention sub-centres, the mean increase in ABER was 6.41 tests/sub-centre (95%CI 4.69, 8.14; p<0.01) and in API was 9.2 cases diagnosed/sub-centre (95%CI 5.18, 13.21; p<0.01). The control sub-centres reported lower increases in ABER (2.84 [95%CI 0.35, 5.34]; p<0.05) and API (3.68 [95%CI 0.45, 6.90]; p<0.05). The control-adjusted post–pre changes in API showed that 5.52 more cases (95%CI 0.34, 10.70; p<0.05) were diagnosed, and a 3.6 more cases (95%CI 0.58, 6.56; p<0.05) were tested per sub-centre in the intervention versus control areas. Larger differences in post–pre changes in API between intervention and control sub-centres were registered in the higher transmission-risk areas compared with the lower risk areas. All the changes were statistically significant.

**Conclusions:**

Intensive intervention activities targeted at improved access to malaria diagnosis and treatment produced a substantial increase in blood examination and case notification, especially in inaccessible, hard-to-reach pockets. CCMP provides insights into how to achieve universal coverage of malaria services through a routine, state-run programme.

## Introduction

In 2016, India accounted for 6% of the global malaria burden and 90% of the malaria cases in the World Health Organization (WHO) South East Asia region [1]. India aims to eliminate malaria by 2030, but there exist significant gaps in malaria surveillance, diagnosis, treatment and control [1]. A major challenge is the substantial heterogeneity in the malaria burden and risk of transmission between and within Indian states with a large diversity in ecotypes and vectors [2].

Responsibility for malaria control is divided between the central and the state governments of India. Technical and operational guidance is provided by the National Vector Borne Disease Control Programme (NVBDCP); the services are provided by the State Vector Borne Disease Control Division through the public health care system. The key elements of India’s malaria control strategy include early case detection and complete treatment (EDCT), based on parasitogical diagnosis of all suspected cases and complete treatment of all confirmed cases, along with vector control measures and surveillance [3].

Odisha, a state in eastern India, has the highest reported malaria burden in the country, contributing 45% of total cases, albeit with only 4% of the Indian land mass and 3% of its population [3]. A large part of Odisha has conditions that are conducive to malaria transmission, such as hilly forested areas with perennial streams, high humidity and medium-to-high rainfall. Malaria control efforts in Odisha were intensified from 2008 onwards, with scaling up coverage of interventions together with active programme management, strong administrative and political commitment as well as substantial state-level financial support. In addition, in 2010 the network of malaria services expanded dramatically with the involvement of village-level female health volunteers, the Accredited Social Health Activists (ASHAs), in the provision of EDCT in malaria endemic areas [4]. Previously, the lowest level of health services was the sub-centre, covering a population of 5,000 people, run by male or female multipurpose health workers.

ASHAs were introduced by the National Rural Health Mission (NRHM) in 2005 to support the public health delivery system. Each ASHA covers a population of about 1,000 people. Starting from 2010, ASHAs were progressively involved in malaria control activities in Odisha. They were trained to diagnose, treat and report their activities to the sub-centre using the standard forms of the NVBDCP. ASHAs were equipped with rapid diagnostic tests (RDTs) and anti-malarial drugs in line with the National Treatment Guidelines. ASHAs started functioning as fever treatment depots, and people suffering from fever were encouraged to seek malaria diagnosis and treatment from them. This led to a dramatic reduction in the malaria burden, especially in the period between 2011 and 2013 [3]. Despite the impressive progress, half of 30 districts in Odisha remained highly malaria endemic, with an annual parasite incidence (API) >10 cases/1000 population [5].

Achieving and maintaining high coverage of the affected population with multiple measures poses serious challenges to the over-stretched health system of one of the most resource-constrained states of India [6,7]. Although malaria services were brought closer to the community, frequent stock-outs of RDTs and treatments at the ASHA level, together with inadequate skills, resulted in sub-optimal access to EDCT [8,9]. Moreover, universal coverage could not be achieved with the ASHA network, as far-flung hamlets, common in malaria-endemic areas in Odisha, did not meet the NRHM population criteria to merit an ASHA. These were served only by ASHAs travelling infrequently from neighbouring, sometimes distant, villages. These remote hamlets faced barriers to timely access to malaria services resulting in a high, though under-reported malaria burden that fuelled the persistent transmission of the disease [10,11]. Weaknesses in the collection, validation, and reporting of malaria data from ASHAs hampered surveillance efforts [8]. There was no system to verify treatment compliance and parasite clearance from the patients’ blood [12,13].

Strong case management is essential as a means of ‘treating the sick’ to reduce the duration of sickness and prevent the disease progressing to severe malaria. However, beyond this purpose, early termination of infections serves, particularly in areas of unstable malaria with low-to-moderate intensities of transmission, as an important means of curtailing the size of the infectious reservoir [14–16]. Strengthening malaria case management system through community-based interventions has been tried out in various other countries such as South Africa, Ethiopia, Myanmar and Cambodia though with variations in the approach and outcomes [15,17–19]. Within India, because a large part of the population in Odisha lives in areas of low-to-moderate endemicity, it is an ideal setting to implement a comprehensive case management system and explore its impact on transmission.

In 2013, the Comprehensive Case Management Project (CCMP) was initiated as an implementation research programme [20], run collaboratively with the national and the state chapter of NVBDCP and National Institute of Malaria Research with support from Medicines for Malaria Venture, Switzerland. CCMP aims to examine the impact of universal and timely access to diagnosis and treatment, as well as improved surveillance, on the transmission of malaria in different settings, against the backdrop of prevailing vector control measures. CCMP also aimed to better define the burden and epidemiological profile of malaria in the area. In this paper we describe the components of CCMP and examine its outcome in the first three years of its implementation.Lessons learnt in terms of rolling-out various components of comprehensive case management and its initial impact on case detection could inform the adoption of similar interventions elsewhere.

## Methods

### Study design and setting

**Fig 1:**
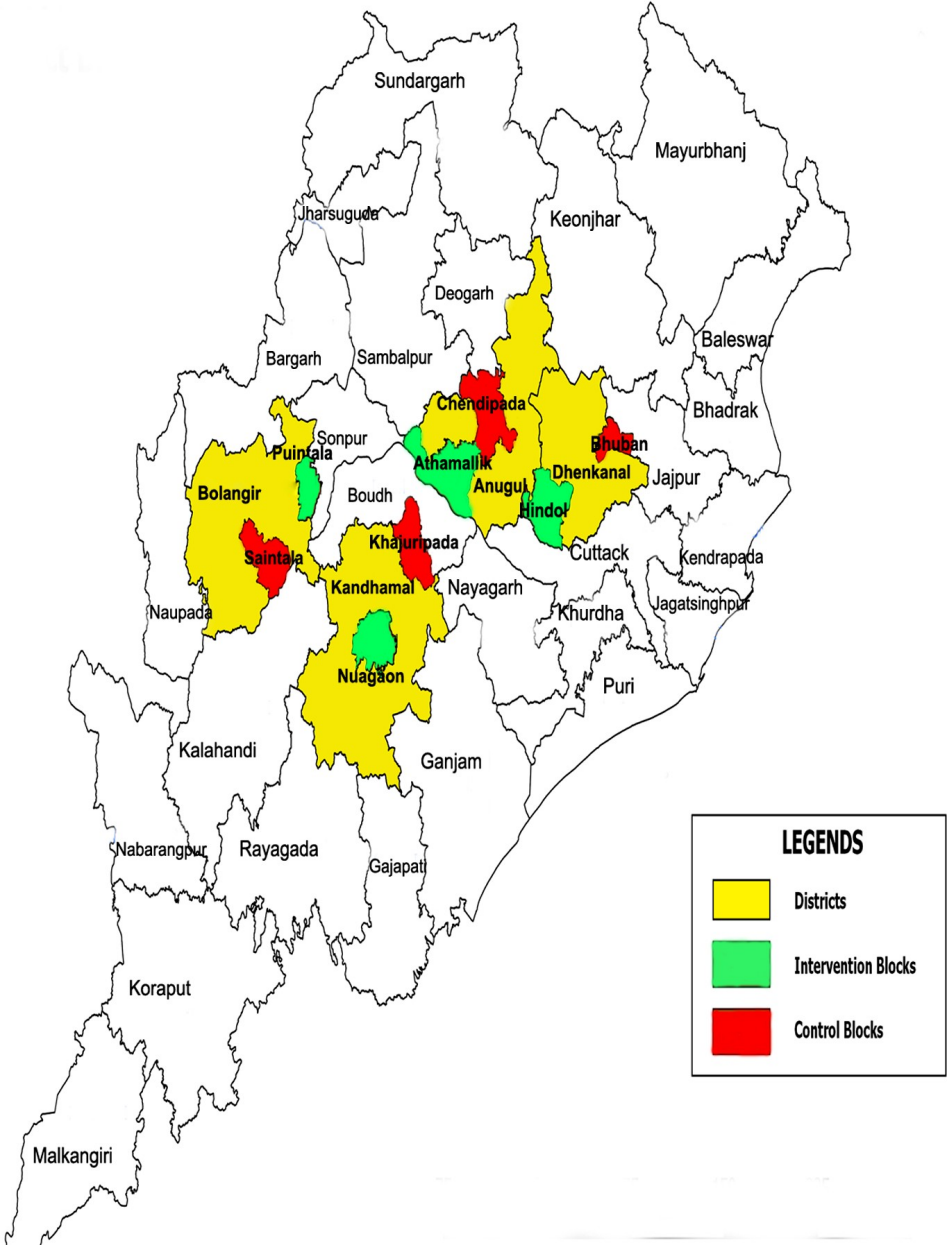
Map of Odisha with the CCMP intervention and control blocks.

**Fig 2:**
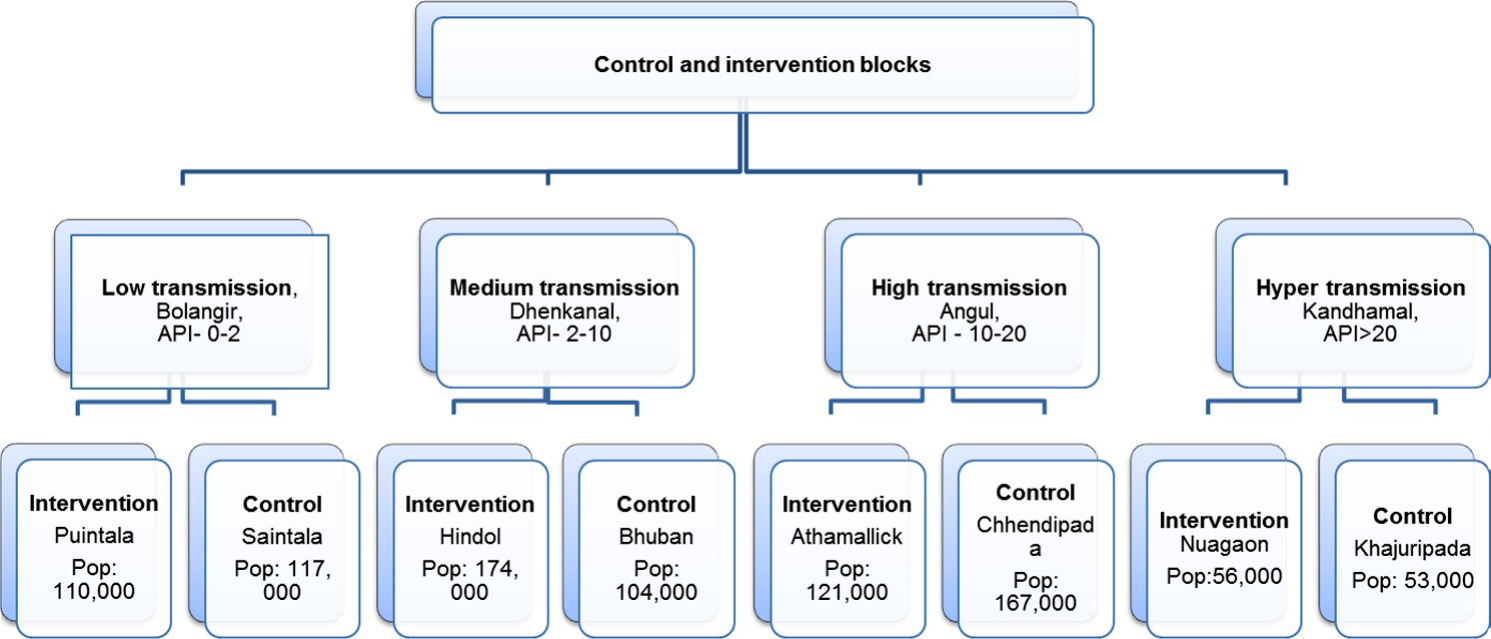
Population details for CCMP intervention and control areas.

CCMP employed a two-arm, quasi-experimental design. Pairs of intervention and control blocks were selected from four districts in Odisha, stratified by transmission intensity (low, medium, high and hyper-endemic) (Fig 1). Each pair of blocks was matched on malaria incidence using routine programme data and selected by a two-stage stratified sampling method (Fig 2). A ‘block’ is the smallest general administrative unit of an Indian district, roughly covering 100,000 inhabitants. The public health system has two more tiers below the block, namely the primary health centre, which covers a few sub-centres, which in turn covers a few villages.

**Fig 3:**
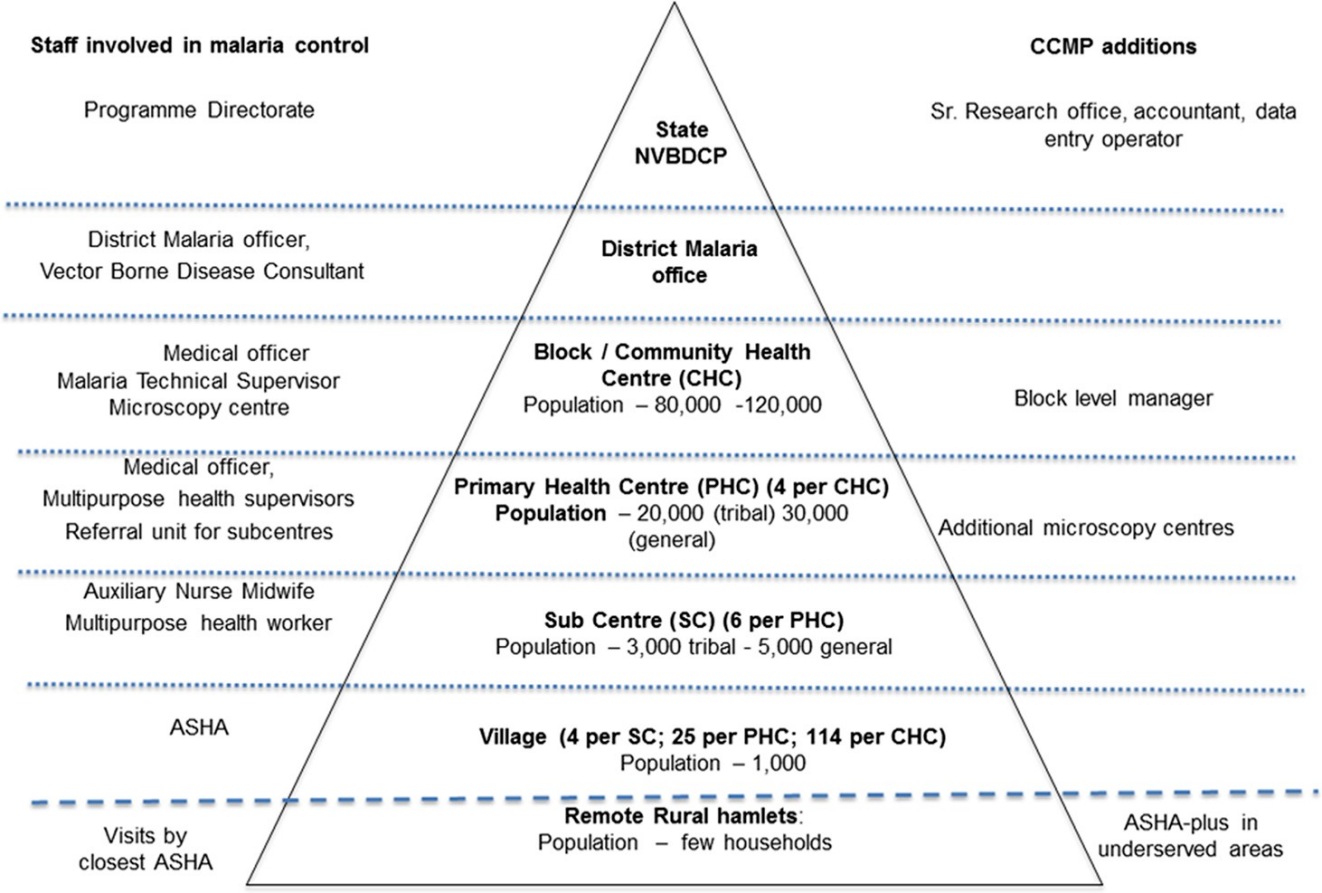
Health service for malaria at different levels of the system.

In the control areas, malaria services were provided within the constraints of the routine programme. In the intervention areas, various measures were undertaken to improve the coverage and quality of malaria services (Fig 3). The supply chain management system was strengthened up to the village level to ensure uninterrupted supply of drugs and diagnostics. In particular, the quantification and supply of RDTs and drugs was calculated taking the caseload, number of service providers and minimum stock levels into consideration instead of only caseload, as in the routine system. Buffer stocks were maintained at the block level in the CCMP areas, rather than at the district level in the routine system. Special attention was paid to ensuring adequate stocks in remote areas before the rainy season, when malaria transmission is at its peak and road access even more difficult.

CCMP established additional microscopy centres at the primary health centre level, to complement the one at the block level under the NVBDCP. The CCMP microscopy centres provide supportive supervision to ASHAs and other providers, in addition to confirming diagnosis and post-treatment parasitological clearance. CCMP appointed block level managers (BLMs) in each intervention area to improve malaria surveillance and facilitate evidence-based action. The BLMs collected the standard NVBDCP forms from all the malaria diagnostic and treatment points at the weekly sub-centre level meetings and ensured the completeness of data. They derived a patient line listing of all positive cases, reassigned patients seeking treatment at higher level facilities to their villages, entered the information into spreadsheets and exported this into DHIS2, an electronic health management information system. Automated monthly report cards were issued with key epidemiological indicators to inform programme activities and permit timely action.

The BLMs identified hard-to-reach villages and hamlets with no resident ASHAs, yet close to sub-centres with a high API. Additional local volunteer-providers (ASHA-plus) were engaged and trained, most of whom were Anganwadi workers (AWW), village-level volunteers of the Women and Child Development Department. The others were mainly village youths involved in local health activities. ASHA-plus providers play the same role as ASHAs–they test all fever cases for malaria, provide confirmed cases with the appropriate treatment and monitor treatment completion. However, in the hyper-endemic area of Kandhamal, as some hamlets comprise small clusters of few households, ASHA-plus providers simply inform ASHAs in the neighbouring village about any fever case in their hamlets. ASHAs were compensated by an incentive payment defined and covered by the NRHM; the ASHA-plus workers are compensated by CCMP.

Focal screening and treatment (FSAT), as a component of CCMP intervention package, was carried out in isolated hard-to-reach villages/hamlets by a team of health workers, ASHAs and ASHA-plus providers using bivalent *P*. *falciparum/P*. *vivax* RDT. Blood slides were also taken for subsequent examination. These areas were purposefully selected based on an analysis of historic data of nearby sub-centres, poorly accessible areas and high malaria transmission risk factors. All positive cases were treated, even if they were asymptomatic.

The skills of all the health workers, including multi-purpose workers and their supervisors, were strengthened through intensive training, refresher courses and supportive supervision. Patient cards, with unique identification numbers, were introduced to track treatment adherence, adverse events, if any, and identify relapses or recurrences. Special attention was paid to identifying primaquine-related adverse events through the development of job aids and training.

### Data collection

Epidemiological data for the control area were derived solely from the routine NVBDCP system. Data for the intervention areas for the years 2011–2012 were based on the routine NVBDCP system and thereafter from the DHIS2 information system. Data related to key transmission risk factors were collected directly from the villages of both control and intervention areas. These data were dichotomized: distance from ASHA/or any other public health facility or provider (≤1 km/>1 km), access to the village by road (good throughout year/poor), proximity to irrigation canal (no/yes), location of village (plains/forested foothills), proximity to natural streams (no/yes). The risk factor data were then aggregated as a proportion of the population of the constituent sub-centres exposed to these factors, as they are the unit for impact analysis. The annual number of fever cases tested for malaria, malaria test-positive cases and mid-year population for each sub-centre was used to calculate the Annual Blood Examination Rate (ABER) and API.

### Statistical analysis

The inputs, processes and outputs of CCMP are presented in Table 1. The sub-centres (n = 77 in the intervention area; n = 72 in the control area) were first characterised by the proportion of their population exposed to the five transmission risk factors. We then obtained a composite risk score for each sub-centre from these five variables using principal component analysis. The first principal component (PC1) explained 59% of the variance in all the risk variables and was then used as a single risk score for the sub-centres [21,22]. The difference in the risk score between the intervention and the control sub-centres was examined using the non-parametric Man Whitney U-test. The differences in the distribution of sub-centres tertiled using risk score across intervention versus control were also tested using chi-square test.

**Table 1:** Key CCMP inputs, process and output indicators in the intervention blocks.

Indicator		2013	2014	2015
CCMP staff deployed	Block level	4	4	4
	Sub-block level	14	14	13
Sub-block level microscopy centres established and functional		13	13	14
Training of health workers	ASHAs trained/re-trained	488	382	383
	Other health staff trained/retrained	240	158	99
ASHA-plus providers	Areas without providers at year start	265	76	43
	Areas without providers at year end	76	43	29
	Anganwadi workers engaged	113	159	134
	Village volunteers engaged	0	56	56
	% trained	100	100	100
Primaquine adverse reaction	Village level providers trained	0	407	0
	Other health staff trained	0	236	0
	Adverse events reported	0	0	0
Patient follow-up	% patient cards issued	45	98	99
	% post-treatment slide confirmation	43	93	97
Output indicators	No. of malaria tests conducted	72,003	109,996	112,788
	No. of positive cases	4,661	11,081	9,963
	% test positivity	6	10	9
	% *P*. *falciparum*	87	76	68
	% *P*. *vivax*	11	20	29
	% Mixed	2	4	3
ASHA and ASHA-plus performance	% tests conducted	43	52	51
	% positive cases detected	52	54	55
Focal screening and treatment	No. of villages covered	2	28	104
	Population of covered villages	299	8,461	24,747
	% population tested for malaria	66	83	78
	% positive	35	6	5
	% asymptomatic out of positives	45	49	68

The primary outcome variables were the sub-centre level ABER and API during the pre- (2011 and 2012) and post-intervention period (2013 to 2015). Only data from passive surveillance was used in this analysis; the FSAT data were excluded. The change in pre- to post-intervention average ABER and API, and the extent to which this change varied between intervention and control, were estimated using difference-in-difference (DID) analysis in a linear regression framework.

The basic DID regression equation used is as follows:

$$Y=\beta_{0}+\beta_{1}x_{1}+\beta_{2}x_{2}+\beta_{3}x_{1}.x_{2}+\varepsilon$$

Where:

*x*_1_ = time period; *x*_1_ = 0, 1 where 0 indicates pre-intervention, and 1 post-intervention.

*x*_2_ = Intervention status; *x*_2_ = 0 indicates the control group, and 1 indicates the intervention group.

*x*_1_.*x*_2_ = indicates the interaction between time period and the intervention status.

Greek letters *β*_0_, *β*_1_, *β*_2_ and *β*_3_ are all unknown parameters to be estimated and *ε* is a random, unobserved ‘error’ term.

The coefficients -

*β*_0_ = intercept.

*β*_1_ = changes in pre and post-intervention group, irrespective of intervention or control.

*β*_2_ = differences between intervention and control, irrespective of pre–post.

*β*_3_ = effect of intervention (difference-in-difference).

The DID regression model was then further adjusted for PC1 score to account for differences in transmission risk across these two arms, if any. Further, whether the project impact varied across different tertiles of transmission risk, was also formally tested using a three-way interaction term in the regression model: time period X intervention status X risk tertile [23]. All analyses were carried out using R v3.0.1 software [24].

### Ethics statement

The study was approved by the Institutional Ethics Committee of ICMR-National Institute of Malaria Research.

We only used aggregated data in our analysis, which was de-facto anonymised. Hence there was no need for informed consent.

## Results

### Improved access to services

The utilization of malaria services in the intervention areas improved as ASHAs and other service providers had the required commodities and skills to diagnose and treat patients at the village level (Table 1). With the progressive engagement of ASHA-plus providers the number of underserved villages/hamlets decreased by 89%, from 265 at the beginning of 2013 to 29 by the end of 2015. ASHAs and ASHA-plus providers tested half of all fever cases and diagnosed and treated 55% of malaria cases at the village level in 2015. The number of malaria tests increased, as did the positivity rate for tests. Notably, the progressive introduction of a bivalent *P*. *falciparum*/*P*. *vivax* RDT in the routine programme in 2013 led to an increase in *P*. *vivax* cases identified. Focal screening and treatment (FSAT) conducted in 104 remote villages, covering 78% of the resident population of 24,747 people, revealed a 5% test positivity rate. Asymptomatic cases accounted for 68% of those who tested positive.

### Descriptive epidemiology

Both intervention and control areas had a similar caseload at the outset based on the malaria incidence as reported by the routine health system. In the post-intervention period (2013–15), the ABER increased versus pre-intervention (2012) by 71% in the CCMP intervention areas, across all transmission settings, from 14 to 24 (Table 2). The increase in ABER in the control areas was considerably less, from 17 in 2012 to 22 in 2015 (29%). There was an increase in the API in the intervention and control areas with an approximately 2-fold increase overall. This increase was particularly evident in the medium and high endemic areas (Table 2).

**Table 2:** Epidemiological overview in CCMP intervention and control areas, 2011–2015.

Outcome	District	Total		Bolangir		Dhenkanal		Angul		Kandhmahal	
	Endemicity			Low		Medium		High		Hyper	
	Status	CCMP	Control	CCMP	Control	CCMP	Control	CCMP	Control	CCMP	Control
	Block			Puintala	Saintala	Hindol	Bhuban	Athamallick	Chhendipada	Nuagaon	Khajuripada
**ABER**	2011	15	18	13	16	10	14	23	18	16	34
** **	2012	14	17	16	14	6	14	18	15	22	34
** **	2013	15	16	15	10	11	12	19	17	21	37
** **	2014	23	21	20	23	18	15	29	21	33	33
	2015	24	22	17	21	19	16	31	23	35	32
**API**	2011	9	8	3	3	2	3	17	10	23	26
** **	2012	10	7	1	1	1	1	16	6	37	32
** **	2013	10	6	2	3	2	1	27	4	14	30
** **	2014	23	9	2	8	9	2	62	10	26	25
	2015	21	14	2	6	10	1	52	16	26	51
**Cases**	2011	4,010	3,629	356	335	347	268	2,067	1,701	1,240	1,325
** **	2012	4,324	3,076	148	123	114	95	1,934	1,071	2,128	1,787
** **	2013	4,661	2,678	216	354	283	76	3,352	644	810	1,604
** **	2014	11,081	4,230	196	936	1,657	163	7,714	1,712	1,514	1,419
	2015	9,963	6,536	194	739	1,704	139	6,461	2,828	1,604	2,830
**% Pf**	2011	91	90	49	35	74	73	96	96	99	99
** **	2012	96	93	59	23	82	65	97	94	100	98
** **	2013	87	93	77	84	75	51	86	93	98	98
** **	2014	76	76	68	71	61	63	77	69	92	90
	2015	68	64	39	55	48	55	71	60	84	72

ABER, Annual Blood Examination Rate per 1,000 population; API, Annual Parasite Incidence per 1,000 population; Pf, *P*. *falciparum*.

### Transmission risk stratification

Although the intervention and control blocks were not matched on their inherent risk for malaria transmission, the transmission risk composite scores, expressed by PC1, differed only marginally (Table 3). There were no significant differences in the distribution of intervention and control sub-centres across the three tertiles of PC1 (Table 3).

**Table 3:** Distribution of risk-stratified sub-centres across CCMP intervention and control.

Transmission risk	CCMP (N = 77)	Control (N = 72)	p value
Median (IQR) score	−0.73 (3.18)	−0.16 (2.15)	0.6
Classification of sub-centres			
High risk	26 (34%)	24 (34%)	0.11
Intermediate risk	20 (26%)	29 (40%)	
Low risk	31 (40%)	19 (26%)	

### Difference-in-difference (DID) analysis

There was a statistically significant increase in the overall ABER in the CCMP intervention sub-centres as well as control sub-centres (Table 4). The post–pre DID estimates in ABER between these two sets of sub-centres indicated a significant effect of the CCMP intervention on increasing ABER (3.6 [95%CI 0.58, 6.56]; p<0.05) (Table 4). There was a significant increase in the API of the intervention sub-centres and also in the control sub-centres (Table 4). The difference in post–pre changes in API indicated a statistically significant increase in API in the CCMP sub-centres versus the controls (5.52 [95%CI 0.34, 10.70]; p<0.05) (Table 4).

**Table 4:** Post−pre differences between CCMP intervention and control sub-centres for ABER and API.

Outcome	CCMP sub-centres post–pre difference (95%CI) *p* value	Control sub-centres post–pre difference (95%CI) *p* value	DID (95%CI) *p* value CCMP vs. control
ABER	6.41 (4.69, 8.14); p<0.01	2.84 (0.35, 5.34); p<0.05	3.6 (0.58, 6.56); p<0.05
API	9.2 (5.18, 13.21); p<0.01	3.68 (0.45, 6.90); p<0.05	5.52 (0.34, 10.70); p<0.05

In the risk-stratified analysis of sub-centres, the difference in post–pre changes in API between intervention and control sub-centres declined consistently with the decline in transmission risk (Table 5). Larger differences in post–pre changes in API between intervention and control sub-centres were registered in the higher transmission-risk areas, whereas relatively smaller differences were registered in the lower risk areas. The statistical test for this intervention–response relationship was statistically significant (p<0.01). The corresponding DID estimates for ABER also varied across the three risk strata, but, there was no tangible intervention–response gradient in this indicator.

**Table 5:** Post−pre differences between CCMP intervention and control sub-centres stratified by transmission-risk categories for ABER and API.

Outcome	Transmission risk	DID Intervention vs. Control (95%CI) p value	p value for trend
ABER	High	7.44 (2.97, 11.91); p<0.01	p<0.01
	Intermediate	0.42 (–6.66, 7.51); p = 0.91	
	Low	2.65 (–0.60, 5.89); p = 0.11	
API	High	13.10 (1.69, 24.45); p<0.05	p<0.01
	Intermediate	5.52 (–4.45, 15.48); p = 0.28	
	Low	0.31 (–1.01, 1.63); p = 0.65	

As a sensitivity analysis, the DIDs were re-estimated using sub-centre-level data by matching the sub-centre pairs by their risk propensity scores (intervention 72 versus control 72, instead of 77 sub-centres in the intervention group in the original analysis). The DID estimates for ABER and API were almost same as those obtained using PC1 adjusted DID estimates (S1 Table).

## Discussion

CCMP improved the utilization of malaria services through training and supportive supervision and by ensuring adequate stocks of RDTs and drugs to diagnose and treat patients at the village level. CCMP also improved access to EDCT by engaging ASHA-plus providers in hard-to-reach villages which had limited access to malaria services under the routine system. This led to a significant increase in people tested for malaria and malaria cases detected, in general, and at the village level, in particular. Efforts to strengthen pharmacovigilance however failed to report any adverse events. This requires further attention and innovation beyond simply training.

CCMP also strengthened the routine information system by checking the completeness of data and improving the quality of data. Although data is routinely collected through the NVBDCP surveillance system, this is often simply used for reporting purposes and is often incomplete. The regular review of the data highlighted many things on which action was taken such as the discovery of the remote hamlets and a boarding school from which children would take malaria to their home villages and has helped avert malaria outbreaks in the low endemic areas. Routine malaria control in most situations lacks of this kind of ‘evidence-based action’ as opposed to blanket operations.

All these activities have led to greater increase in passive malaria testing (ABER) and API in the intervention areas in comparison to the control blocks, as established from the analysis of pooled data. This rise in these two important indicators in the intervention area over the control may be causally ascribed to the CCMP activities [3,25]. When the sub-centres–our units of analysis–were disaggregated into three categories by their transmission risks, higher control-adjusted post−pre surges in API were more evident in high transmission risk sub-centres than their low-risk counterparts and a clear intervention–response gradient could be observed [22]. The likely reason for the greater impact of CCMP in higher transmission risk units might be the higher parasite burden in these areas and hence more ‘unreached’ cases in the pre-CCMP era, which responded more dramatically to CCMP strategies, whereas, the lower risk units had a lighter burden of malaria, which led to more moderate results there.

The greater impact of CCMP in terms on higher ABER and API in the less accessible, high endemic areas strengthens the inference we draw regarding the causal linkage between CCMP and the post−pre surge in malaria case notification, in view of CCMP’s focus on increasing overall access to EDCT, with special emphasis on remote areas. CCMP highlighted the extent to which ‘routine’ data underestimates the true burden of disease because of poor surveillance. As API is the key indicator used by the NVBDCP to identify areas for vector control measures, these areas fall under the radar as they are caught in a cycle of limited access to malaria services, under-detection, under-reporting, under-served and hence continued malaria transmission.

We hypothesize that this initial surge in case notification achieved by CCMP activities should eventually lead to a reduction in parasite burden, particularly in the previously unreached communities in the intermediate term. This in turn should lead to a decline in the actual incidence of malaria and hence notification from these populations, despite sustained high levels of malaria testing. Good compliance with treatment for most cases, confirmed by the parasite clearance by microscopic examination, might also facilitate this decline [26]. The full dataset will be analysed to test this hypothesis once the programme draws to its close at the end of 2017.

The research programme had some inherent weaknesses owing to unavailability of pre-intervention data and data from the control block on selected indicators, such as the level of health services at which patients were diagnosed and treated, to establish the effects of some interventions. Although there was *a priori* matching of the intervention and control blocks based on epidemiological indicators, this did not take transmission risk factors into account. Therefore, although it is likely that some unmeasured systematic differences existed between them, which could not be accounted for in the analysis, it is unlikely to have confounded our conclusions. Although routine programme data was used for the analysis, any errors in the data are mostly random and non-differential, especially in the pre-CCMP era, and unlikely to have greatly affected our conclusions. Vector control measures were carried out in both control and intervention areas in line with the NVBDCP policies and any differences probably had only a small effect on the results obtained.

In conclusion, CCMP revealed the extent of the gap in access to malaria diagnosis and treatment in the prevailing health system and the extent to which the malaria burden is underestimated, particularly in high endemic areas. CCMP improved the utilization and coverage of the routine malaria programme and provides insights into how to achieve this through the routine programme. CCMP also highlighted the value of improved surveillance and the need for more malaria officers who are trained to analyse data and take action based on it. As we expect the malaria burden in the state will decrease with effective interventions, the challenge now is to focus on areas with a large malaria parasite reservoir, owing to poor surveillance, which perpetuates perennial and intense transmission. Thus, improving access to vector control measures as well as early diagnosis and treatment is critical to reduce the malaria burden which traps people in poverty and ill health. As India moves towards the elimination of malaria, CCMP is providing valuable lessons to accelerate this process.

## Supporting information

S1 Table (DOCX)
