# Supplementary material for: Improved access to early diagnosis and complete treatment of malaria in Odisha, India
Source: PLoS One. 2019 Jan 2;14(1):e0208943. doi: 10.1371/journal.pone.0208943 (PMC6314604; doi:10.1371/journal.pone.0208943)
Supplement: S1 Table — (DOCX) [file pone.0208943.s001.docx]

**S1 Table Post−pre differences between CCMP intervention and control sub-centres matched with propensity risk scores for annual blood examination rate (ABER) and annual parasite incidence (API)**

| **Outcome** | **Intervention sub-centres post–pre difference**  **(95%CI) *p* value** | **Control sub-centres post–pre difference**  **(95%CI) *p* value** | **DID CCMP vs. control (95%CI) *p* value** |
| --- | --- | --- | --- |
| ABER | 6.43 (4.46, 8.39); *p*<0.01 | 2.84 (0.35, 5.34); *p*<0.05 | 3.583 (0.41, 6.75); *p*<0.05 |
| API | 8.10 (3.61, 12.59); *p*<0.01 | 3.68 (0.37, 6.98); *p*<0.05 | 4.43 (–1.13, 9.99); *p*=0.12 |

DID, difference-in-difference
